# Genetic or pharmacological disruption of the MSH3 Y245/K246 IDL binding pocket slows CAG repeat expansion

**DOI:** 10.1093/narmme/ugag031

**Published:** 2026-06-05

**Authors:** Rob Goold, Jasmine Donaldson, Florence Gidney, Philip Goff, Joseph Hamilton, Marwa Elmasri, Lucy Coupland, Michael Flower, Sarah J Tabrizi

**Affiliations:** Huntington’s Disease Centre, Department of Neurodegenerative Disease, UCL Queen Square Institute of Neurology, University College London, London WC1N 3BG, United Kingdom; Dementia Research Institute at UCL, London WC1N 3BG, United Kingdom; Huntington’s Disease Centre, Department of Neurodegenerative Disease, UCL Queen Square Institute of Neurology, University College London, London WC1N 3BG, United Kingdom; Dementia Research Institute at UCL, London WC1N 3BG, United Kingdom; Huntington’s Disease Centre, Department of Neurodegenerative Disease, UCL Queen Square Institute of Neurology, University College London, London WC1N 3BG, United Kingdom; Dementia Research Institute at UCL, London WC1N 3BG, United Kingdom; Huntington’s Disease Centre, Department of Neurodegenerative Disease, UCL Queen Square Institute of Neurology, University College London, London WC1N 3BG, United Kingdom; Dementia Research Institute at UCL, London WC1N 3BG, United Kingdom; Huntington’s Disease Centre, Department of Neurodegenerative Disease, UCL Queen Square Institute of Neurology, University College London, London WC1N 3BG, United Kingdom; Dementia Research Institute at UCL, London WC1N 3BG, United Kingdom; Huntington’s Disease Centre, Department of Neurodegenerative Disease, UCL Queen Square Institute of Neurology, University College London, London WC1N 3BG, United Kingdom; Dementia Research Institute at UCL, London WC1N 3BG, United Kingdom; Huntington’s Disease Centre, Department of Neurodegenerative Disease, UCL Queen Square Institute of Neurology, University College London, London WC1N 3BG, United Kingdom; Dementia Research Institute at UCL, London WC1N 3BG, United Kingdom; Huntington’s Disease Centre, Department of Neurodegenerative Disease, UCL Queen Square Institute of Neurology, University College London, London WC1N 3BG, United Kingdom; Dementia Research Institute at UCL, London WC1N 3BG, United Kingdom; Huntington’s Disease Centre, Department of Neurodegenerative Disease, UCL Queen Square Institute of Neurology, University College London, London WC1N 3BG, United Kingdom; Dementia Research Institute at UCL, London WC1N 3BG, United Kingdom

## Abstract

Recent genetic studies have shown somatic expansion of the CAG repeat is the key process driving Huntington’s disease (HD) pathogenesis. Recognition of insertion-deletion loops (IDLs), lesions prone to form within the CAG repeat, by Mutsβ (MSH3/MSH2) is thought to be the primary event in the expansion process. This starts a cascade that leads to error-prone repair and incorporation of additional CAG units into the repeat. *In vitro* data shows MSH3 binds IDLs through a DNA-binding pocket formed by MSH3 residues Y245/K246. In this study, we investigated the significance of this DNA-binding motif in CAG repeat expansion using cell lines harbouring long, unstable *HTT* CAG repeats. Genetic disruption of the MSH3 Y245/K246 motif significantly reduced DNA interaction, exhibited MMR deficiency in a frameshift mutator assay, and abrogated repeat expansion in a U2OS cell line expressing mutant *HTT* exon 1. Pharmacological blockade of this site using a small molecule targeting the DNA-binding pocket similarly reduced DNA binding and repeat expansion in a U2OS cell line. Crucially, this molecule also slowed CAG repeat expansion in medium spiny neurones derived from HD patient-iPSCs. Targeting of the MSH3 IDL binding pocket may represent a possible therapeutic strategy.

## Graphical Abstract

**Graphical Abstract:**
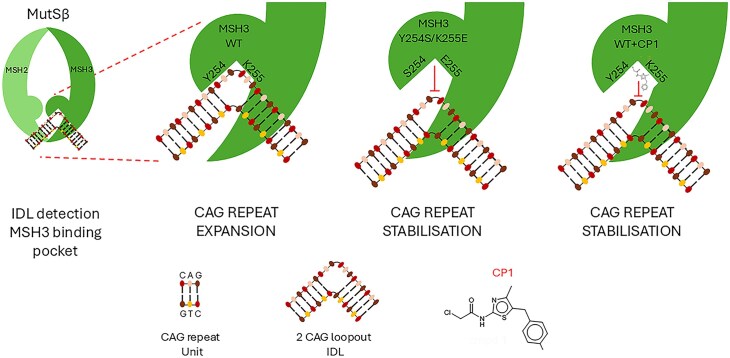

## Introduction

Huntington’s disease (HD) is a devastating genetic neurodegenerative disorder characterized by progressive deterioration of movement, cognition, and mental health. The root cause of HD is an unstable and expanded CAG trinucleotide repeat within the *HTT* gene [1]. This region typically encodes a polyglutamine (polyQ) tract of ~20 glutamines in the general population. However, a repeat length of 36 CAGs or more is associated with disease, with longer repeats correlating with earlier disease onset and increased severity.

The CAG repeat is known to be somatically unstable, meaning it can expand during an individual’s lifetime, leading to very long repeats (exceeding 1000 CAGs) observed in post-mortem brain tissue of HD patients [2, 3]. This expansion is tissue-specific, with more pronounced increases in repeat length found in brain regions primarily affected in HD, particularly the striatum [2]. Interestingly, experimental HD models with CAG repeat lengths in the 40s, typically associated with human disease, often fail to exhibit a clear phenotype. This has led to the hypothesis that a significant expansion of the trinucleotide repeat, generating a longer polyQ sequence in the huntingtin protein, is essential for disease manifestation. A proposed threshold for cellular toxicity is greater than 150 repeats [4, 5]. The expanded polyQ region in the mutant huntingtin protein (mHtt) gains a toxic function, disrupts various cellular processes, and ultimately leads to cell death [1]. Therefore, somatic instability (SI), or the expansion of the CAG repeat, is a critical pathogenic process in HD.

Genetic studies have implicated DNA repair proteins as crucial modifiers of HD [6]. Genome-wide association studies (GWAS) have identified FAN1, a DNA nuclease involved in interstrand crosslink (ICL) repair and replication fork dynamics [7, 8], as a major regulator of HD age at onset (AAO) [9, 10]. Subsequent research has demonstrated that FAN1 slows SI in both cell and mouse models, suggesting this activity underlies its modulatory role [11, 12]. The GWAS analysis also identified components of the DNA mismatch repair (MMR) pathway, specifically MSH3, MLH1 PMS1, and PMS2, as regulators of HD progression [9, 10]. Earlier mouse studies had already pointed to MMR as a driver of both HD phenotypes and repeat expansion [13, 14], providing a plausible mechanism for their role in the disease. These findings highlight that DNA damage response (DDR) proteins can either protect against or exacerbate the HD mutation through their modulation of CAG repeat length [6].

MMR is generally a protective mechanism, scanning the genome for base pair mismatches or small insertion-deletion loops that can arise during DNA replication and transcription [15]. MMR is initiated by one of two heterodimeric MutS homolog complexes: MutSα (MSH2/MSH6) and MutSβ (MSH2/MSH3). MutSβ (MSH2/MSH3) favours small, extra-helical loops (insertion-deletion loops or IDLs), while MutSα (MSH2/MSH6) prefers single base mismatches. The recognition of an appropriate lesion by either MutS complex triggers the recruitment of MutL protein heterodimers, forming ternary complexes. MLH1 is a common component in all MutL heterodimers, partnering with PMS2 (MutLα), PMS1 (MutLβ), or MLH3 (MutLγ) [15]. Upon activation by interaction with MutS and PCNA, MutLα creates endonucleolytic incisions in the DNA flanking the mismatch [16], while MutLγ nicks the opposing strand [17]. PMS1 lacks a nuclease domain, so MutLβ lacks endonuclease activity, and its specific function remains unknown. The lesion is subsequently removed by exonuclease EXO1, followed by resynthesis and ligation facilitated by DNA polymerase δ and LIG1 [15].

Less is understood about the mechanisms driving SI in the presence of a mutant CAG repeat, where MMR paradoxically adopts a detrimental role. In this context, aberrant attempts by MMR components to repair DNA lesions lead to the inclusion of additional CAG units into the repeat. While the precise mechanism remains unclear, this process is dependent on MutSβ, a dependency reflected in GWAS findings and studies in HD cell and animal models [18–23]. In contrast, MutSα plays a limited role in repeat expansion [22, 23]. *In vitro* studies have shown that MutSβ binds with high affinity to IDLs, including those formed by short [1–3] CAG loopouts [24–26]. These IDLs are prone to form in highly repetitive sequences such as the *HTT* CAG repeat, a phenomenon attributed to strand slippage, polymerase stalling, and the formation of R-loops during transcription or replication. The recognition of these lesions by MutSβ and the subsequent recruitment of downstream factors like MutL complexes and EXO1 result in the aberrant repair pathway that ultimately drives repeat expansion. Notably, all MutL factors contribute to CAG repeat expansion [14, 27], and knocking down any single component (MLH1, PMS1, PMS2, or MLH3) slows expansion in an *ex vivo* HD model [28]. Thus, MSH3/MutSβ, through its DNA binding and protein–protein interactions, plays a central role in SI [15].

MSH3 interacts with MSH2 to form the functional MutSβ heterodimer. This interaction is mediated by two primary regions: an N-terminal region (amino acids 126–250) and a carboxy-terminal region (amino acids 1050–1128) [29]. MSH3 binds to MLH1 and PCNA via overlapping motifs located near the N-terminus. PCNA binds through a conserved PIP box spanning residues ^21^QAVLSRFF^28^, while MLH1 binds to the SRFF MIP box sequence contained within the PIP box. *In vitro* data suggest that these two proteins compete for occupancy of these sites [30]. Additionally, MSH3 possesses intrinsic ATPase activity, which is essential for MMR and has been linked to repeat expansion [31].

MSH3's binding to IDLs is mediated by a binding pocket within its mismatch binding domain (MBD). The importance of this site was initially identified in yeast systems [32]. Structural studies have since shown that tyrosine 245 interacts with the base pair immediately upstream of the IDL, and lysine 246 makes contact with the phosphodiester backbone of the IDL [25, 33]. Substitutions at these positions (Y245S/K246E) in MSH3 abolish the interaction of MutSβ with IDLs, hairpin loops, and G4/R-loops [34, 35], all structures implicated in trinucleotide repeat expansion.

In this study, we investigated the significance of the MSH3 Y245/K246 DNA-binding motif in CAG repeat expansion using cell lines harbouring long, unstable *HTT* CAG repeats. Genetic disruption of this motif significantly reduced DNA interaction and abrogated repeat expansion in a U2OS cell line expressing mutant *HTT* exon 1. This MSH3 mutant also exhibited MMR deficiency in a frameshift mutator assay. Furthermore, pharmacological inhibtion of this site using a small molecule targeting the DNA binding pocket [36] similarly reduced DNA binding and repeat expansion in a U2OS cell line. Critically, this molecule also slowed CAG repeat expansion in medium spiny neuron (MSN)-enriched cultures derived from HD patient-induced pluripotent stem cells (iPSCs).

## Materials and methods

### U2OS cell culture and manipulation

U2OS cells featuring FRT sites introduced into the genome were a gift from Prof. John Rouse (University of Dundee, Scotland). Routine culture of this line, transduction with a lentiviral *HTT* exon 1 construct harbouring 118 CAG repeats (LV HTT exon 1 118Q), and expansion assays were previously described [12]. *MSH3* knockout (KO) and introduction of transgenes into the FlpIn site were performed as previously described [12, 21]. Myc MSH3 6a cloned into the pcDNA 5.1 FRT/TO-Puro vector was generated by VectorBuilder. Mutations were introduced into this backbone by SDM using the QuickChange XL kit (Agilent, CA, USA). Two lines of U2OS MSH3 KO cells were isolated and complemented with the myc MSH3 forms. These were used in parallel for all the experiments described in the manuscript. For example, expansion assays were performed in quadruplex, with two independent timecourses from each KO clone or complemented derivative. Transgene expression was induced by adding doxycycline (dox) direct to cell media routinely at 0.1 ng/ml for endogenous expression levels or at 1 ng/ml for overexpression studies. When using the MSH3 E976A mutant, 1 ng/ml doc was used to induce endogenous levels of MSH3. Fresh media and dox were added every 2–3 days. Dox was included in some parallel assays using U2OS MSH3 KO cells. This made no difference to the expansion rate observed as previously reported [12]. CP1 (2-chloro-N-[4-methyl-5-[(4-methylphenyl)methyl]-1,3-thiazol-2-yl]acetamide) was purchased from ENAMINE and added to cell media from a 20 mM stock dissolved in DMSO. Media with fresh CP1 was replaced every 2 days. Toxicity of CP1 was assessed using an MTT viability assay following CP1 exposure for two cell passages (10 days) as previously described [12, 21].

### Human iPSC line maintenance and differentiation

The QS5.1 125 CAG iPSC line (IRB number: CENSOi019-A) was derived previously from fibroblasts from a 7-year-old female paediatric HD individual with fully informed parental consent and ethical approval from the local ethics committee with good clinical practice (GCP) compliance. The fibroblasts were reprogrammed by Sendai-based methods at Censo Biotechnologies. This iPSC line originally had a repeat sequence containing 125 uninterrupted *HTT* CAG repeats, followed by a single CAACAG cassette. Prior to differentiation, HD iPSCs were maintained in Stem Flex (Gibco) on Geltrex (1:100, Gibco)-coated plasticware and passaged at 70%–80% confluency using ReLeSR (STEMCELL technologies).

Differentiation into MSN-enriched neuronal cultures was achieved following the 36-day protocol as described in Arber *et al*. [37], which involves the promotion of lateral ganglionic eminence fate via activin A treatment, followed by terminal differentiation into striatal neurones. After differentiation, the MSN-enriched cultures were treated continuously from day 36 (referred to as baseline, or day 0) with CP1 or vehicle (DMSO) as control, and wells were harvested every 21–28 days to monitor CAG repeat instability. Toxicity of CP1 was assessed using an MTT (Sigma–Aldrich, 475989) viability assay following CP1 exposure for 84 days. For immunocytochemistry, cells were fixed with 4% PFA for 15 min, washed with PBS, and permeabilized with ethanol (15 min at room temperature, RT). The cells were blocked for 1 h in 5% BSA, 1% goat serum, and 0.5% Triton X-100 (block buffer, BB), then primary antibody was added overnight in BB. After four washes of 10 min with PBS-T (0.1% Triton X-100), samples were incubated with secondary antibodies in BB and Hoechst counterstain for 2 h RT. Following four further 10-min PBS-T washes, samples were prepared for microscopy the following day with 90% glycerol and 0.5% N-propyl gallate in 0.1 M Tris pH 7.4. Images were acquired on an Opera Phenix (PerkinElmer/Revvity) from 10 random fields in three wells from each experiment. Z-stacks of 10 layers were combined for each image. Primary antibodies used were βIII-tubulin (ab107216), DARPP32 (ab40802), and FOXP1 (ab32010) from Abcam; MAP2 (NB300-213, Novus); and purified mouse anti-Ki-67 (BD Pharmingen, 550609). AlexaFluor secondary antibodies were from Invitrogen.

### Immunoprecipitation, ChIP, and biotinylated oligo pull-down

Extracts for immunoprecipitation (IP) were prepared as previously described [12, 21], and IP was done overnight at 4°C using anti-MSH3 or anti-MLH1 antibodies (BD Biosciences) coupled to protein A/G magnetic beads or anti-myc tag Dynabeads (Pierce). Biotinylated oligos were purchased from IDT and annealed by heating to 95°C for 5 min, then slow cooling to room temperature. Oligo sequences were as follows: CGACTTCCGGTAGCACGTAGCACTGCTGCGCCACGAACTGCACTCTAGGC and GCCTAGAGTGCAGTTCGTGGCGCAGCAGCAGCAGTGCTACGTGCTACCGGAAGTCG.

These were coupled to MyOne Streptavidin T1 Dynabeads via a 5′ biotin moiety conjugated to the top strand according to the manufacturer’s instructions (Pierce) using 75 ng of annealed oligo per µl of beads. Cell extracts were prepared by lysing cells in TBS pH 7.4 with 1% Triton X-100 and protease inhibitors (Sigma). Lysates were sonicated for 30 s at 40% power in a ultrasonic processor 500 W probe sonicator and then centrifuged at 20 000 × *g* for 3 min. Supernatants were adjusted to 2 mg/ml and used as inputs for pull-downs, using 800 µg protein and 20 µl beads for each. Input and IP or pull-down fractions were prepared for immunoblotting as previously described [12, 21]. Antibodies used were MSH3 rabbit polyclonal (Proteintech, used for ChIP and some Westerns); MSH3 mouse monoclonal, and MLH1 mouse monoclonal and MSH6 mouse monoclonal (BD Biosciences); MSH2 and PCNA rabbit (Cell Signaling Technology); and an actin mouse monoclonal (Sigma). Uncropped blots are included in the Supplementary data (Supplementary Fig. S6). ChIP analysis was performed with the EZ-Magna ChIP Kit as previously described [12, 21] using CAG set 3 (forward: CCTTCGAGTCCCTCAAGTCCTT, reverse: CGGCTGAGGCAGCAGCGGCTGT) and GFP primers (forward: AAGGGCGAGGAGCTGTTCA, reverse: CTGCCGTCCTCGATGTTGT) as detailed in the figure legend. Results were expressed as fold increases relative to U2OS MSH3 KO ChIP or control samples, as indicated in the main text.

### SI measurements in U2OS cells (fragment analysis)

**Figure 1.**
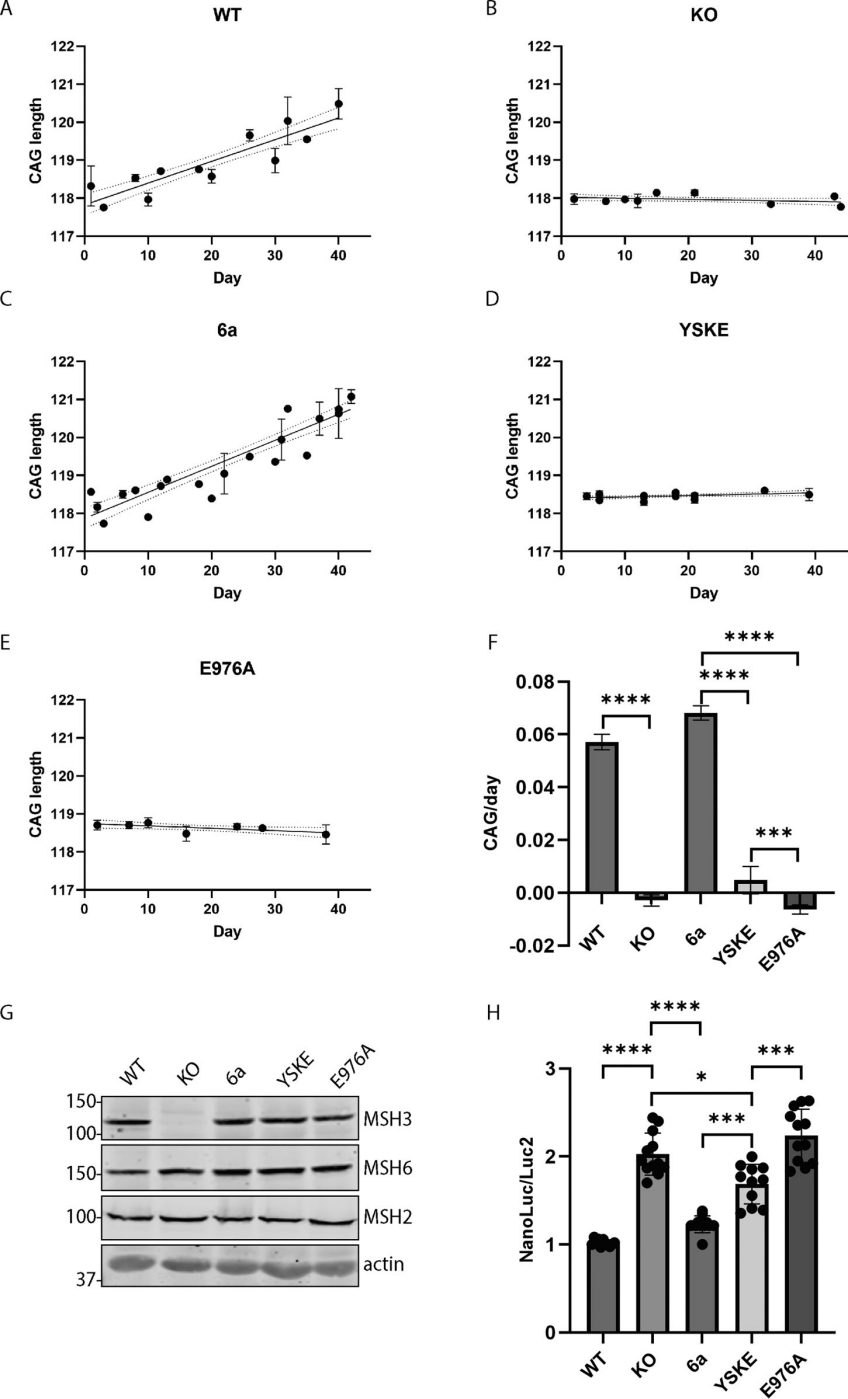
Genetic blockade of the MSH3 IDL binding pocket disrupts MMR function. (**A**–**E**) U2OS WT, MSH3 KO cells, or KO cells complemented with myc MSH3 6a, YSKE, or E976A constructs were transduced with LV *HTT* exon 1 118 CAG. Repeat size was monitored over 40 days in culture using fragment analysis. Data from two KO clones or complemented cells derived from them were run in duplicate. WT cells were measured in triplicate. Simple linear regression curves fitted to the data along with the 95% confidence interval (dotted lines) are shown (mean ± SD). (**F**) Histogram shows regression analysis of expansion curves in panels (A–E) (mean ± standard error, ***P < .005, ^****^P < .001). WT/myc MSH3 6a and MSH3 KO/myc MSH3 E976A expansion rates were not significantly different. (**G**) Western blot showing expression of MutS proteins in WT, MSH3 KO, and KO cells complemented with myc MSH3 6a, YSKE, or E976A constructs. Complemented cells were treated with 0.1 ng/ml dox, except the E976A line, in which 1 ng/ml dox was used. (**H**) U2OS WT, MSH3 KO, or MSH3 KO cells complemented with myc MSH3 6a, YSKE, or E976A were transduced with an EMAST frameshift reporter construct. This 16-tetranucleotide-repeat construct measures MSH3-dependent MSI in cells, assayed by measuring NanoLuc signal normalized to a constitutive Luc signal. Results are expressed as NanoLuc/Luc2 normalized to WT cells (mean ± SD, *P < .05, ***P < .005, ^****^P < .001). WT and myc MSH3 6a NanoLuc/Luc2 signals were not significantly different.

U2OS cells transduced with the HTT exon 1 118Q virus show relatively rapid expansion with an easily identifiable peak with a clear modal value on fragment analysis profiles. Therefore, modal CAG length provides an accurate metric to assess expansion. Expansion assays and fragment analysis were performed as previously described [12, 21] using GeneMapper v6 software (Thermo) to align the chromatographs. To calculate modal CAG repeat length, GeneMapper data were exported and analysed with a custom R script, available at https://michaelflower.org/. For expansion assays (Fig. 1A–E and Supplementary Fig. S1C), data represents two clones per genotype, with KO clones or complemented KO clones run in duplicate. WT cells were measured in triplicate. In Fig. 5A–C, data from three expansion assays were run for each condition, with each measured twice. All data points included in the analysis are shown in the Supplementary data (Supplementary Fig. S5A–C).

### SI measurements in MSN-enriched cultures (long-read repeat sizing)

Expansion of the CAG repeat in striatal cultures was assayed using long-read repeat sizing. These cultures show slower and more complex expansion than HTT exon 1 118Q-transduced U2OS cells, often developing multimodal peak profiles. Simple modal CAG length measures do not fairly reflect the overall expansion of the CAG repeat in these cells. Hence, the instability index metric was used to measure the whole spectrum of repeat expansion observed in MSN-enriched cultures. Genomic DNA (25 ng per reaction) was amplified using locus-specific primers flanking the HTT exon 1 CAG repeat (MF20230707_20 assay). Each primer incorporates a unique 16-bp barcode, enabling asymmetric dual-barcode sample indexing from the first amplification cycle. Primers were designed in SNP-free, haplotype-conserved regions to ensure universal amplification without allele-specific bias. The ∼3 kb amplicon minimizes preferential amplification of shorter alleles by reducing the relative length difference between expanded and non-expanded alleles to ~5%.

PCR was performed in 20 µl reactions using Platinum SuperFi II DNA Polymerase Master Mix (Invitrogen, 12368010) with 0.3 µM of each barcoded primer under the following conditions: 98°C for 30 s; 28 cycles of 98°C for 10 s, 60°C for 10 s, and 72°C for 2 min; followed by 72°C for 5 min. Products were verified by 1% agarose gel electrophoresis, quantified by Qubit dsDNA HS assay, normalized to equimolar concentrations, and pooled. Pooled amplicons were purified using 1.8× AMPure XP beads (Beckman Coulter, A63881).

SMRTbell libraries were prepared from pooled amplicons using the SMRTbell Prep Kit 3.0 (PacBio, 102-141-700). Amplicons underwent DNA damage repair and end-repair/A-tailing, followed by ligation of indexed SMRTbell adapters. Libraries were treated with Exonuclease III and VII to remove unligated fragments, purified using SMRTbell cleanup beads, and sequenced on the PacBio Revio platform to generate high-fidelity (HiFi) circular consensus reads.

### Bioinformatic analysis

HiFi reads were demultiplexed by dual-barcode assignment using Ophelia (Ophelia: 10.5281/zenodo.18888576) and analysed using the Duke pipeline (Duke: 10.5281/zenodo.18888560). Reads were aligned to the amplicon reference sequence and CAG repeat tract length was measured for each read. Reads were assigned to alleles based on repeat length, and the modal repeat length was determined as the most frequently observed value within predefined ranges (normal: 0–35 CAG; expanded: ≥36 CAG).

SI metrics were calculated relative to the modal repeat length of the baseline timepoint. The instability index was defined as the mean absolute deviation from the modal length; the expansion index as the mean deviation for reads above the modal length; and the expansion ratio as reads above the modal length divided by reads at the modal length. For analyses of MSN-enriched cultures presented in Fig. 5, the instability index was used as the primary quantitative summary of somatic CAG repeat expansion, as it captures shifts across the full repeat-length distribution over time. In Fig. 5E–G, three independent parallel cultures were run for each condition (vehicle, 2.5 µM CP1, and 5 µM CP1), and each was measured twice. All data points included in the analysis are shown in the Supplementary data (Supplementary Fig. S5D–F).

### EMAST reporter assays

Cells were transduced with a lentiviral EMAST frameshift reporter construct [38] and cultured for five days, including 0.1 ng/ml dox in complemented cell cultures. NanoLuc and Luc2 signals were measured using the NanoGlo kit (Promega) and luciferase assay substrate (Abcam), respectively. Results are expressed as NanoLuc/Luc2 normalized to WT cells.

### Quantification and statistical analysis

CAG expansion time courses were analysed by linear regression and slopes statistically compared by one-way ANOVA. Multiple comparisons were corrected for with a false discovery rate of 5%. The Brown–Forsythe test was routinely used to check for homogeneity of variance. All statistical information can be found within figure legends.

## Results

### Generating a cell system to study MSH3 function in CAG repeat expansion

We have previously used U2OS cells transduced with an HTT exon 1 118Q construct to study repeat dynamics and shown these cells support expansion in culture, adding approximately one CAG to the repeat every 18–20 days (Fig. 1A and F and Supplementary Fig. S1A) [12]. Following MSH3 KO, these cells did not support CAG repeat expansion in line with findings from other HD models (Fig. 1B and F and Supplementary Fig. S1B) [21]. The MSH3 KO cells were complemented with a WT myc tagged MSH3 6a construct expressed under a dox inducible promoter (the MSH3 6a isoform is the most common allele—Ref sequence NC_000005.10 [39]). Maintaining myc MSH3 6a construct expression at similar levels to endogenous MSH3 with 0.1 ng/ml dox rescued repeat expansion to rates seen in U2OS WT cells (Fig. 1C and F and Supplementary Fig. S1C and F). Increasing dox to 1 ng/ml led to eight-fold overexpression of myc MSH3 6a and increased the expansion rate of the CAG repeat in the HTT exon 1 118Q construct to above that seen in U2OS WT cells, approaching the levels seen in U2OS FAN1 KO cells [12] (Supplementary Fig. S2A–D). These data demonstrate the importance of MSH3 in driving CAG repeat expansion and show the U2OS system can provide a test bed to assess the role of MSH3 functions in CAG repeat expansion.

To test whether IDL binding by MSH3’s DNA-binding motif plays a role in CAG repeat expansion, we introduced Y254S/K255E substitutions into the 6a construct, referred to here as YSKE (corresponding to Y245S/K246E in the canonical human MSH3 sequence described in [25]). We also generated an MSH3 E976A Walker B ATPase mutant as a comparator. This mutation is well characterized, is known to disrupt MMR activity, and has been associated with reduced repeat expansion [31]. Unlike the MSH3 6a construct, these mutants did not promote repeat expansion effectively (Fig. 1D–F and Supplementary Fig. S1D and E), despite expression at comparable levels (Fig. 1G and Supplementary Fig. S1F). Although neither mutant drove expansion efficiently, expansion rates observed in E976A and YSKE cells were significantly different. In fact, myc MSH3 E976A cells show a small decrease in repeat size over time that is significant (slope different from zero, *P* = .031), whereas myc MSH3 YSKE cells show a small but significant increase in repeat size (slope different from zero, *P* = .0141).

As an independent measure of MMR activity in cells expressing these mycMSH3 constructs, we performed a frameshift mutator assay designed to measure MSH3 activity. This assay is based on a 16-repeat tetranucleotide sequence that is prone to elevated microsatellite alterations at selected tetranucleotide repeats (EMAST) linked to a reporter in an expression cassette. When introduced into a cell, it is prone to alterations in size, either by addition or deletion of an AAAG four-nucleotide unit. This shifts the construct into frame with the reporter, in this case a NanoLuc sequence. Maintenance of the repeat region relies largely on MSH3 activity, and deficiency in function increases EMAST, which leads to a reporter expression [37]. This EMAST reporter is preferentially sensitive to MutSβ (MSH2/MSH3) function and is less dependent on MutSα (MSH2/MSH6), making it well suited to assess MSH3-specific mismatch repair activity in this system.

In U2OS WT cells a baseline NanoLuc signal is detected after 5 days, indicating a relatively low level of frameshifts in the reporter construct, as would be expected (Fig. 1H) [37]. MSH3 KO cells show a consistent increase in NanoLuc activity, roughly doubling the signal over the course of five days in culture. Importantly, complementation of the MSH3 KO cells with myc MSH3 6a, expressed at endogenous levels, restores baseline EMAST activity (Fig. 1H). This demonstrates the MSH3 dependence of the EMAST reporter. In contrast, cells expressing the myc MSH3 E976A ATPase dead form show high levels of EMAST, with similar NanoLuc signal to MSH3 KO cells. Cells expressing myc MSH3 YSKE mutant also show EMAST deficiency, but the NanoLuc signal does not reach the levels in KO or E976A mutant lines (Fig. 1H).

### MSH3 molecular interactions underlie cell phenotypes

**Figure 2.**
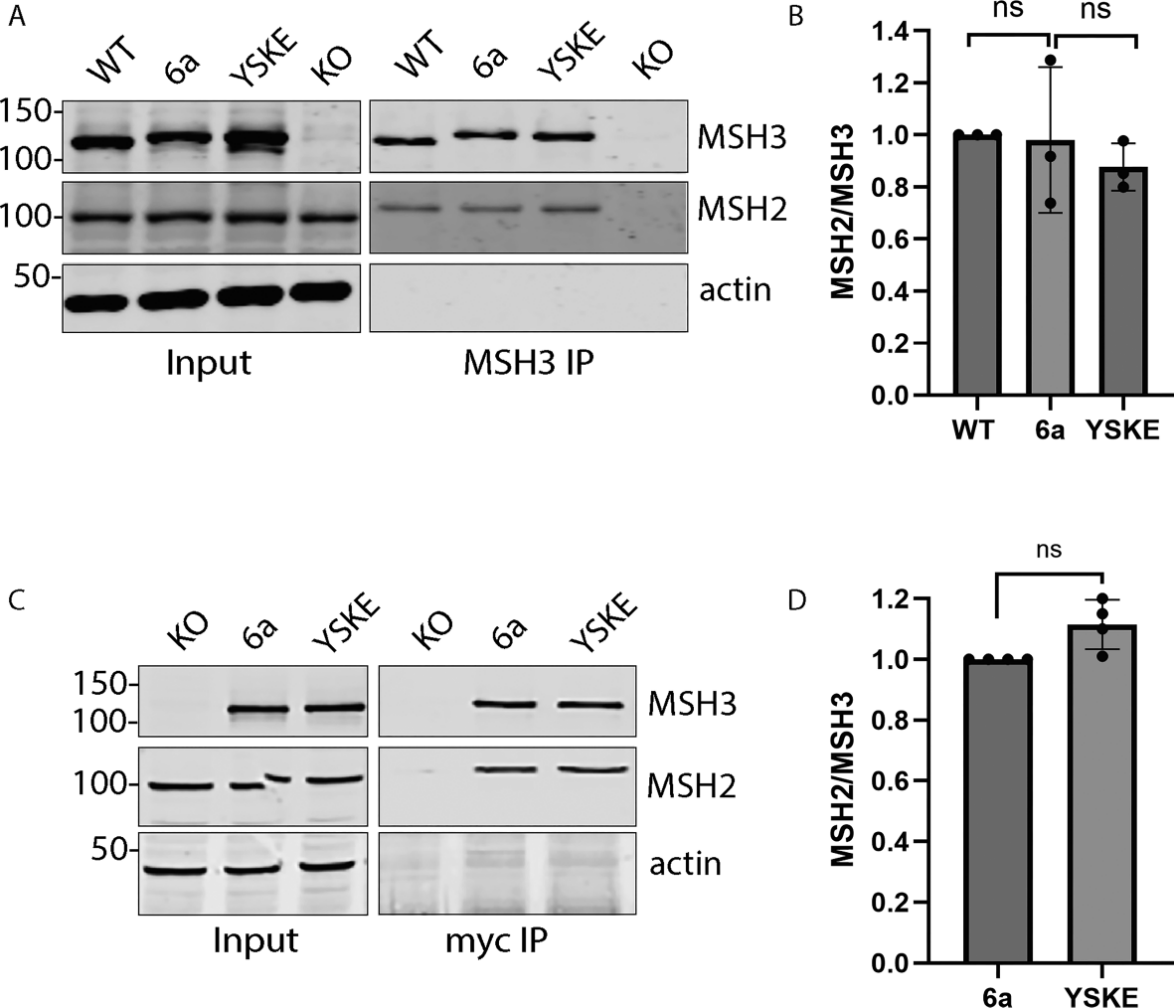
MutSβ heterodimerization in WT and myc MSH3-complemented cells. (**A**) U2OS cell extracts from WT, MSH3 KO, or KO cells complemented with myc MSH3 6a or YSKE constructs treated with 0.1 ng/ml dox were incubated O/N with MSH3 antibodies and protein A/G magnetic beads. Beads were isolated and washed with a magnetic device. Input (5%) and proteins eluted from the beads were immunoblotted with the indicated antibodies. Actin is included as a loading control. (**B**) Quantification of MSH2/MSH3 binding from IP experiments normalized to WT MSH3 is shown in the histogram (mean ± SD, ns = non-significant). Similar levels of MSH2 were recovered in the MSH3 IP fraction from both WT MSH3, myc MSH3 6a, and YSKE samples. (**C**) U2OS cell extracts from MSH3 KO or KO cells complemented with myc MSH3 6a or myc MSH3 YSKE were incubated O/N with myc magnetic beads. Transgenes were overexpressed by ~8-fold with 1 ng/ml dox. Beads were isolated and washed with a magnetic device. Input (5%) and proteins eluted from the beads were immunoblotted with the indicated antibodies. Similar levels of MSH2 were recovered in the myc IP fraction from both MSH3 6a and YSKE samples. Actin is included as loading control. (**D**) Quantification normalized to MSH3 6a is shown in the histogram (mean ± SD, ns = non-significant).

These assays show interesting MSH3-related cell phenotypes. To understand the mechanism underlying these phenotypes, we have studied the molecular interactions of WT MSH3 and the myc tagged 6a and YSKE constructs. MutSβ complex formation was studied using an IP approach. Using 0.1 ng/ml dox induction and MSH3 antibodies allowed a comparison of the activity of WT MSH3 and myc MSH3 6a or YSKE transgenic forms at equivalent expression levels (Fig. 2A). The procedure immunoprecipitated robust levels of MSH3 from all input fractions. MSH2 co-fractionates with MSH3 in all the IP samples, indicating the MutSβ complex formed normally with transgenic MSH3 proteins (Fig. 2A and B). The specificity of the procedure is demonstrated by use of an MSH3 KO line as control.

One advantage of the U2OS system is that the level of transgene expression can be precisely controlled. As we showed previously, myc MSH3 6a overexpression accelerates CAG repeat expansion (Supplementary Fig. S2C and D). Expansion is generally believed to be dependent on MutSβ-driven MMR, which in turn requires ternary complex formation between DNA, MutSβ, and MutL components [15]. To see if MSH3 overexpression is accompanied by increased MutSβ heterodimerization and MutL binding, we performed MLH1 IPs. In these experiments, cell extracts were prepared for IP from myc MSH3 6a cells treated with 0.1 or 1 ng/ml dox. IPs were performed with MLH1-specific antibodies and input or IP fractions were immunoblotted for MSH3, MSH2, and MLH1 (Supplementary Fig. S1E). Increased MSH3 expression was accompanied by a small increase in MSH2 expression but a bigger increase in MSH3/MSH2 co-IP with MLH1, suggesting an increase in MutSβ/MutL complex formation (Supplementary Fig. S1E and F). MSH3 levels in the cell extracts and IP fraction increased proportionally more than MSH2, reflecting the incorporation of most of the MSH2 into MutSα, which is the dominant MutS form present in cells.

This indicates that overexpression of MSH3 6a results in an increase in the active MMR complex capable of enhancing CAG repeat expansion that retains the molecular interactions that drive the process. We took advantage of this to look at myc MSH3 protein and DNA interactions in more detail by overexpressing the transgenes in our cells; this facilitates the detection of binding partners. Firstly, we looked at myc MSH3 protein interactions using myc beads to pull down the tagged proteins. The IP fractions from the MSH3 6a and MSH3 YSKE forms contained equivalent levels of MSH2, showing the Y254S/K255E substitutions did not affect MutSβ heterodimerization (Fig. 2C and D).

**Figure 3.**
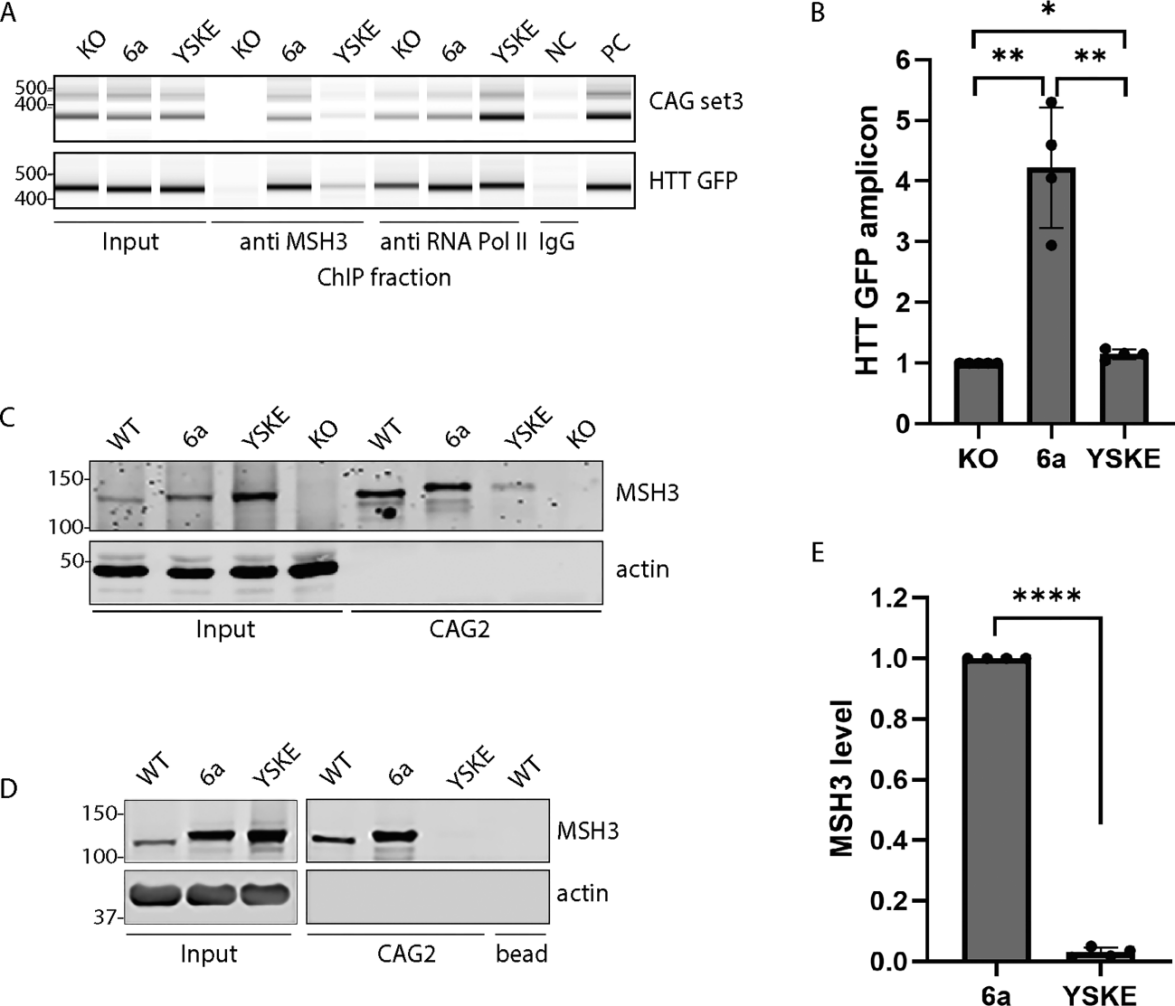
MSH3 DNA interactions in WT and myc MSH3-complemented cells. (**A**) Cell extracts from HTT exon 1 118Q transduced MSH3 KO cells or KO cells complemented with myc MSH3 6a or myc MSH3 YSKE were prepared for ChIP analysis and immunoprecipitated with anti-MSH3, RNA PolII, or control non-specific mouse IgG antibodies (NC). DNA from input and ChIP fractions was purified and probed with primers targeting the *HTT* exon 118Q CAG repeat in the construct (CAG set 3) or GFP primers downstream from this in the insertion cassette (HTT GFP). Input (5%) and ChIP fractions were analysed using TapeStation apparatus and software. MSH3 ChIP fractions from YSKE mutant contain less CAG repeat and HTT GFP DNA. Note that the myc MSH3 cells were treated with 1 ng/ml dox, which induces ∼8× MSH3 overexpression relative to WT cells. Plasmid DNA was amplified alongside the ChIP fractions as a positive control (PC). (**B**) Quantification of HTT GFP levels in ChIP fractions normalized to MSH3 KO is shown (mean ± SD, **P < .01, *P < .05). (**C**) U2OS cell extracts from WT cells, KO cells, or KO cells complemented with myc MSH3 6a or myc MSH3 YSKE and treated with 0.1 ng/ml dox were prepared. These were incubated O/N with magnetic streptavidin beads conjugated to CAG2 oligos or empty beads. Input (5%) and eluted proteins (CAG2) were immunoblotted with the indicated antibodies. Similar levels of MSH3 were recovered in the pull-down fractions from WT and myc MSH3 6a, indicating the WT and myc tagged MSH3 forms have similar activity. Pull-downs from myc MSH3 YSKE extracts contained only low levels of MSH3. MSH3 KO cell extracts (KO) were used as controls. (**D**) U2OS cell extracts from WT cells or KO cells complemented with myc MSH3 6a or YSKE were prepared and incubated O/N with CAG2 oligos immobilized on streptavidin magnetic beads. Beads were isolated and washed with a magnetic device. Input (5%) and eluted proteins (CAG2) were immunoblotted with the indicated antibodies. Unconjugated beads were used as controls (WT sample only). Note that the myc MSH3 cells were treated with 1 ng/ml dox, which induces ∼8× MSH3 overexpression relative to WT cells (compare lanes WT and 6a). (**E**) Quantification of MSH3 levels in CAG2 oligo pull-down fractions normalized to myc MSH3 6a is shown (mean ± SD, ^****^P < .001).

DNA interactions were initially assessed by ChIP using a specific antibody to isolate MSH3 and associated DNA. Extracts from MSH3 KO and myc MSH3 cells were prepared that gave eight-fold overexpression of myc MSH3 forms (Supplementary Fig. S1A and B). This allowed unequivocal detection of CAG repeat and control HTT GFP DNA in the ChIP fractions (Fig. 3A). ChIP fractions from myc MSH3 6a contained more CAG repeat and HTT GFP DNA compared to background levels detected in ChIP fractions prepared from KO cells or those isolated with a non-specific IgG control antibody. In contrast, the myc MSH3 YSKE mutant showed reduced DNA association, either with the CAG repeat or downstream HTT GFP DNA (Fig. 3A). Despite this, low levels of DNA were consistently detected in YSKE ChIP tractions. Quantification of the HTT GFP amplicon, chosen as it gave a clearer signal, showed significant differences in the levels of DNA in the myc MSH3 6a ChIP fractions compared to KO and myc MSH3 YSKE (Fig. 3B). myc MSH3 YSKE ChIP fractions contained significantly more DNA than KO ChIP fractions (Fig. 3B). Control ChIP fractions made using RNA polymerase II antibodies contain similar amounts of DNA in all samples.

**Figure 4.**
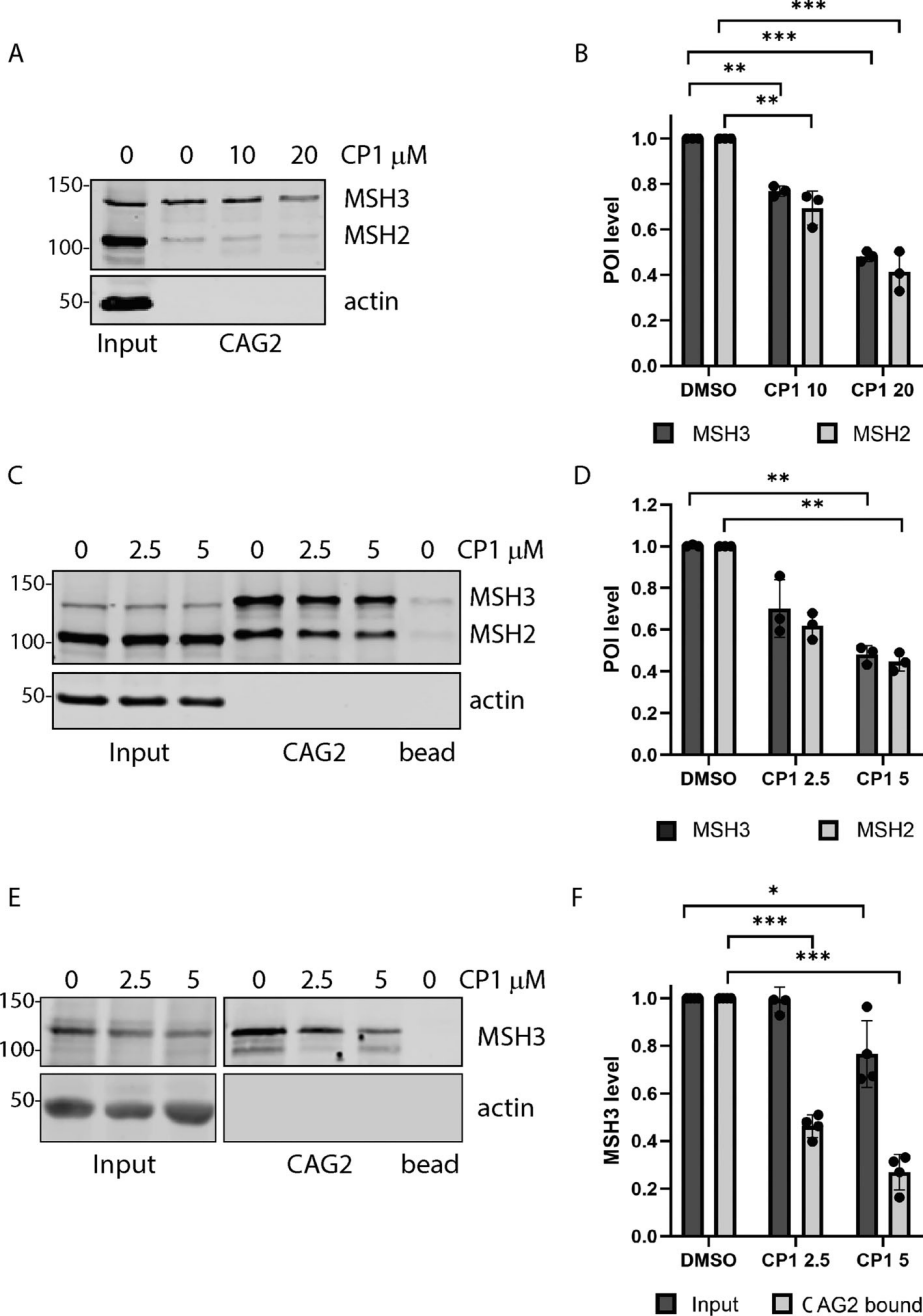
Pharmacological inhibition of the MSH3 IDL binding pocket reduces DNA interactions. (**A**) U2OS cell extracts from WT cells were treated with vehicle (0) or the indicated CP1 concentrations for 1 h prior to incubation with CAG2 oligos immobilized on streptavidin magnetic beads. Beads were isolated and washed with a magnetic device. Input (5%) and eluted proteins (CAG2) were immunoblotted with the indicated antibodies. (**B**) Quantification of MSH3 and MSH2 levels in CAG2 oligo pull-downs normalized to DMSO control is shown (mean ± SD, **P < .01, ***P < .001). (**C**) U2OS WT cells were treated for 24 h with vehicle (0) or CP1 at the indicated concentrations, and cell extracts were prepared and incubated with CAG2 oligos immobilized on streptavidin magnetic beads as described in panel (A). MSH3 and MSH2 recovery in the bead fraction was reduced by CP1 treatment. Unconjugated beads were included as a control. (**D**) Quantification of MSH3 and MSH2 levels in CAG2 oligo pull-downs normalized to vehicle-treated cells is shown (mean ± SD, **P < .01). (**E**) Differentiated HD iPSC-derived MSN-enriched cultures were treated with vehicle (0) or CP1 (2.5 and 5 µM), and cell extracts were prepared and CAG2 oligo-binding assays performed as in panel (A). (**F**) Quantification of MSH3 levels in the input fraction and CAG2 oligo pull-downs normalized to vehicle-treated cells is shown (mean ± SD, *P < .05, ***P < .005).

To confirm these data, we also performed affinity pull-downs of MSH3 from cell extracts using immobilized biotinylated DNA oligos as bait. We selected a 50-mer sequence with a two-CAG loopout inserted in the 5′–3′ strand (referred to as CAG2 oligos), mimicking the likely DNA lesion targeted by DNA repair apparatus during SI. WT MSH3 and myc MSH3 6a were detected in the pull-downs, binding with apparent high affinity to the CAG2 oligo (Fig. 3C). MSH2 was also detected in the pull-downs, indicating that MutSβ is binding to the CAG2 oligo (Supplementary Fig. S3A and Fig. 4). Proportionally less of the MSH2 input was pulled down compared to MSH3. This is likely because most of the MSH2 is associated with MSH6, which doesn’t bind to the oligo under the conditions used for these experiments. (Supplementary Fig. S3A). Low levels of myc MSH3 YSKE were detected in pull-downs fractions, showing the mutation prevents effective interaction with the DNA oligos (Fig. 3C).

Similar results were obtained when pull-downs were performed from cells overexpressing MSH3 transgenes (Fig. 3D). Quantification showed a significant decrease in the DNA binding of the MSH3 YSKE mutant relative to the 6a form and confirms the DNA binding deficiency shown by ChIP (Fig. 3E).

### A small molecule targeting the MSH3 IDL binding pocket reduces DNA interaction

To confirm the importance of the MSH3 IDL binding site in CAG repeat expansion and test its potential as a therapeutic target, we have used a recently identified small molecule that binds close to Y254/K255 and inhibits DNA binding [36]. This molecule, 2-chloro-N-[4-methyl-5-[(4-methylphenyl)methyl]-1,3-thiazol-2-yl]acetamide (referred to as CP1 hereafter), was one of a library of cysteine-targeting molecules tested. Consistent with its effect on MSH3 activity, CP1 was shown to bind irreversibly to cysteine 252, whose side chain protrudes into the DNA-binding pocket of MSH3.

To verify target engagement in our system, we added CP1 to WT U2OS cell extracts for 1 h and assessed MSH3 binding to CAG2 oligos. This procedure showed dose-dependent inhibition of MSH3 and MSH2 CAG2 oligo binding (Fig. 4A and B). Next, we added CP1 to live cells and assessed target engagement *in situ*. At CP1 concentrations above 10 µM cell toxicity was observed. However, viability assays showed lower CP1 concentrations were well tolerated by the cells (Supplementary Fig. S4A). Therefore, CP1 was added to the live cells at 2.5 and 5 µM. Cells were treated overnight, then extracts were prepared for CAG2 oligo pull down. Immunoblotting shows robust MSH3 and MSH2 binding to the oligo following vehicle (DMSO) addition. MSH2 levels relative to the input fractions were proportionally less compared to MSH3. Importantly, dose-dependent inhibition of MSH3/MSH2 binding was observed at 2.5 and 5 µM CP1 (Fig. 4D). Given the reduced concentration of CP1 used, inhibition was more effective after cell addition than adding CP1 directly to lysates, possibly because of the longer exposure time (Fig. 4A and C).

ChIP experiments were also performed to test target engagement (Supplementary Fig. S3B). FAN1 KO cells^12^ harbouring the LV HTT exon 1 118Q insert were used. This allowed the interaction of MSH3 with the expanded CAG repeat to be assessed. These were treated with vehicle or 5 µM CP1 and prepared for ChIP. Endogenous MSH3 was immunoprecipitated using specific antibodies, and DNA was probed with CAG repeat and HTT GFP primers as described earlier (Supplementary Fig. S3B). Low levels of DNA were detected in DMSO-treated ChIP fractions. This low level of DNA is typical of endogenous MSH3 ChIP in our hands and probably reflects both the relatively low levels of MSH3 in the cells and the transient nature of the MutSβ DNA association. In particular, the long allele of the 118 CAG insert was hard to detect, a consistent problem with the ChIP procedure, especially with cells harbouring very long CAG repeats. This reflects the difficulty in amplifying the CAG repeat as it lengthens and the fact that the ChIP protocol requires DNA to be fragmented, meaning longer DNA sequences are more likely to be broken during preparation. For these reasons, GFP primers targeting a site downstream of the CAG repeat have been included in the analysis. Although CP1 treatment consistently reduced the level of CAG repeat and downstream HTT GFP DNA detected in the ChIP fractions, quantification of the effect was difficult due to the low signal. By contrast, control ChIP experiments using an RNA polymerase II antibody contained robust levels of DNA, which were not affected by CP1 treatment (Supplementary Fig. S3B). Together, these data show CP1 is capable of inhibiting MSH3 DNA interactions at concentrations well tolerated by U2OS cells.

To test the applicability of such compound in a post-mitotic, disease-relevant cell type, CP1’s target engagement was also assessed in HD iPSCs carrying an expanded *HTT* CAG repeat (>125 CAG) [40]. HD iPSCs were differentiated to striatal cultures enriched for MSNs, a neuronal subtype known to be vulnerable in HD. These cultures typically form mixed cell populations that have a high neuronal content (βIII-tubulin+ >95%, MAP2+ >90%), of which roughly one third are MSNs (FOXP1+ and DARPP-32+ >35%), and contain a low level of proliferating cells (Ki67+ ∼5%) (Supplementary Fig. S4C and D). Long-term CP1 treatment had a minimal effect on cell viability or culture content (Supplementary Fig. S4B–D) but did result in a small decrease in MSH3 levels in the cells at 5 µM (Fig. 4E and F). In line with U2OS cell data, MSH3 interaction with the CAG2 oligo was reduced by 2.5 and 5 µM CP1 treatment (Fig. 4E and F). At 5 µM, CP1 MSH3 oligo binding was ~25% of control levels, much larger than the reduction in MSH3 expression (MSH3 levels ∼75% of control).

### A small molecule targeting the MSH3 IDL binding pocket slows CAG repeat expansion

**Figure 5.**
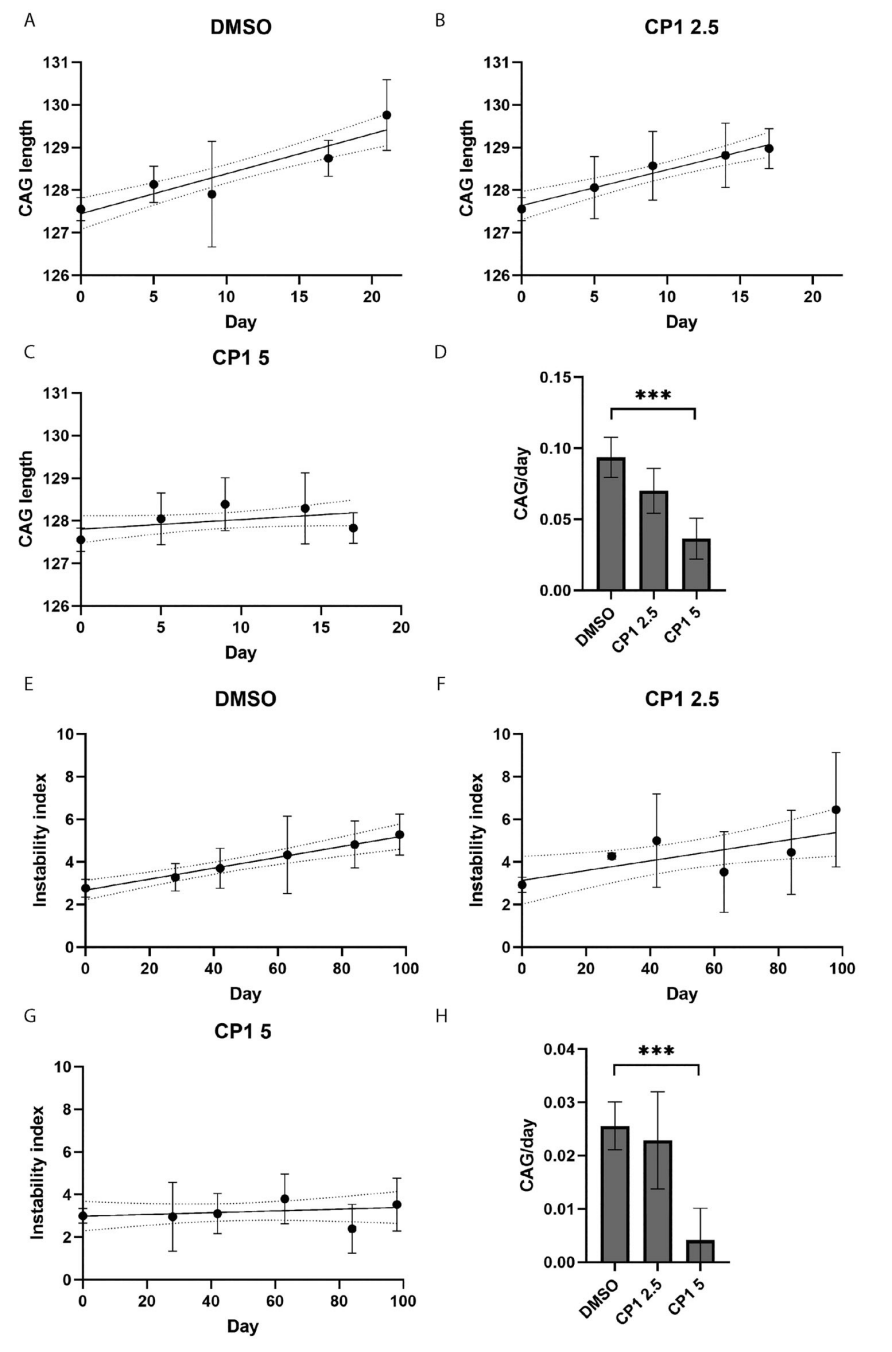
Pharmacological inhibition of the MSH3 IDL binding pocket slows CAG repeat expansion. U2OS FAN1 KO cells previously transduced with LV HTT exon 1 118 CAG were cultured in the presence of vehicle (DMSO) or CP1. Repeat size was monitored over 17 or 21 days in culture using fragment analysis. Vehicle-treated cells (**A**) show an increase in CAG repeat size over time in culture. CP1 treatment at 2.5 µM (**B**) and 5 µM (**C**) progressively reduced CAG repeat expansion rate. Three independent expansion time courses were run in parallel for each condition. Simple linear regression lines fitted to the data are shown along with the 95% confidence interval (dotted lines, mean ± SD). (**D**) Histogram shows regression analysis of expansion curves in panels (A–C) (mean ± standard error, ***P < .005). (**E**) Time course showing *HTT* CAG repeat expansion over time in HD iPSC-derived MSN-enriched cultures treated after differentiation with vehicle (DMSO), CP1 2.5 µM (**F**), or CP1 5 µM (**G**). Day 0 represents the first day of treatment of differentiated cells. Three independent expansion time courses were run in parallel for each condition and measured using long-read repeat sizing of the endogenous *HTT* CAG repeat. Simple linear regression lines fitted to the data are shown along with the 95% confidence interval (dotted lines, mean ± SD). (**H**) Regression analysis of MSN time courses shown in panels (E–G), derived from long-read instability indices (mean ± standard error, ***P < .005). Together, these data show dose-dependent slowing of somatic CAG repeat expansion by CP1 across dividing (U2OS) and post-mitotic (MSN-enriched) systems.

To test the effect of pharmacological inhibition of MutSβ DNA binding on CAG repeat expansion, we treated U2OS FAN1 KO cells transduced with the HTT exon 1 118Q insert with CP1 at 2.5 and 5 µM. These cells had been cultured with the exon 1 construct for 3 months, during which time the repeat had expanded to 127–128 CAGs, compared to freshly transduced cells, which size to roughly 118 CAGs (Fig. 1). These cells were chosen because of the fast expansion rate which reduces the time required in culture to see significant changes in CAG length. The cells were cultured with vehicle (DMSO) or CP1 for up to 21 days and sampled every 4 or 5 days during that time. Genomic DNA was purified from each sample and CAG repeat length was monitored using fragment analysis. DMSO-treated cells expanded at one CAG repeat unit added per 9–10 days in line with previously published data [12]. CP1 treatment slowed expansion at both 2.5 and 5 µM (Fig. 5A–C and Supplementary Fig. S5A–C), showing dose dependency roughly in line with the target engagement (Figs 5D and 4D). Although significance was not reached at 2.5 µM CP1, 5 µM treatment significantly slowed expansion relative to vehicle.

We next looked to assess the effect of pharmacological blockade of MutSβ DNA binding in a non-dividing system. We utilized the HD iPSCs carrying an expanded *HTT* CAG repeat (>125 CAG) described earlier [40]. HD iPSCs were differentiated to striatal cultures enriched for MSNs, a neuronal subtype known to be vulnerable in HD (Supplementary Fig. S4C and D). Differentiated cells were treated with media containing vehicle (DMSO) or CP1 for 98 days, with media changes every 3–4 days. Minimal toxicity was observed with this compound at 5 µM over 84 days (Supplementary Fig. S4B). CP1 treatment at 2.5 and 5 µM progressively reduced CAG repeat expansion rate in differentiated MSNs-enriched cultures compared to vehicle (Fig. 5E–G and Supplementary Fig. S5D–F). Slowing was significant at 5 µM CP1 (Fig. 5H).

## Discussion

Recent genetic studies have shown somatic expansion of the CAG repeat is the key pathogenic process driving HD onset and progression [5]. Recognition of IDLs, lesions prone to form within the CAG repeat, is thought to be the primary event in the expansion process. *In vitro* experiments suggest this is mediated by MutSβ (MSH3/MSH2), and a wealth of evidence supports this MMR pathway as a major driver of SI and, by extension, HD pathogenesis [9, 10, 28, 41]. This makes MSH3 a protein of great interest both in terms of the mechanism of action at the CAG repeat and as a potential therapeutic target. To study MSH3 function, we have developed a U2OS cell model in which MSH3 KO and complementation through a FlpIn site allow endogenous MSH3 function and the function of our introduced 6a (a myc tagged MSH3 transgene with WT sequence) and mutant MSH3 transgenes to be compared. In addition, stable introduction of an HTT exon 118Q construct with a long CAG repeat [12] allows MSH3/CAG repeat interactions to be studied.

One important feature of this model is the ability to precisely regulate transgene expression through the TetOn system [12]. This allows myc MSH3 6a expression at a range of levels. At endogenous levels, expression of myc MSH3 6a restores MutSβ-driven MMR activity and CAG repeat expansion (Fig. 1A and C). Protein and DNA interactions of the myc MSH3 6a are also similar to the endogenous protein (Figs 2A and B and 3C). Interestingly, overexpression of the transgene led to an increase in MutSβ formation, MLH1 binding, and CAG repeat expansion above that seen in WT cells, approaching the rate in FAN1 KO cells (Supplementary Fig. S2) [12]. This shows that overexpression of MSH3 results in an increase in active MMR complex formation that is capable of enhancing CAG repeat expansion, even in the presence of endogenous FAN1. This supports the idea that the expansion rate is determined by the balance between opposing factors, those that promote expansion (MSH3/MMR) and those that slow it down (FAN1), as suggested by recent *in vitro* work [42].

Having established a model in which transgenic MSH3 expression can recapitulate *MSH3* gene function, we have looked in detail at the IDL binding pocket in MSH3 and its effect on CAG repeat expansion. Structural studies have identified tyrosine 245 and lysine 246 as key residues making close contact with DNA lesion [25]. Substitutions at these positions (Y245S/K246E) in MSH3 abolish the interaction of MutSβ with IDLs, hairpin loops, and G4/R-loops [34, 35] *in vitro*, suggesting this site could play a role in CAG repeat expansion. These corresponding substitutions were introduced into our myc MSH3 6a construct (as Y254S/K255E). Myc MSH3 YSKE protein expression and MSH2 interactions were not obviously affected by the substitutions (Fig. 2). MSH3 YSKE DNA binding was markedly reduced, consistent with published *in vitro* data [34, 35] (Fig. 3). Thus, despite normal MutSβ formation, DNA binding was compromised.

Inefficient DNA interaction is likely to underlie the MMR deficiency seen in myc MSH3 YSKE-complemented cells. However, this did not reach the levels seen in KO or MSH3 E976A cells (Fig. 1H). Importantly, CAG repeat expansion was impaired in MSH3 YSKE cells (Fig. 1D). The HTT exon 118Q CAG repeat showed only minimal increases in length over the 40-day time course. Similar cells carrying myc MSH3 E976A construct, a well-characterized Walker B ATPase mutant, also failed to support CAG repeat expansion and showed a tendency to small reductions in repeat length. We have seen this phenomenon previously in cells with compromised MSH3 function [40]. These small differences between the E976A and YSKE forms in both EMAST and CAG repeat expansion assays may indicate some residual MMR activity is present in the DNA binding mutant, which could be related to low levels of residual DNA binding detected (Fig. 3A and C).

To further study the role of the MSH3 IDL binding site in CAG repeat expansion, we used a recently identified small molecule that binds close to Y254/K255. This molecule, referred to here as CP1, binds irreversibly to cysteine 252 within the MSH3 IDL binding pocket and inhibits DNA interaction [36]. Using a CAG2 oligo-binding assay and ChIP, we confirmed CP1 reduces MSH3 DNA binding *in vitro* and showed its inhibitory activity is retained in live cells (Fig. 4 and Supplementary Fig. S3). Remarkably, CP1 treatment of U2OS HTT exon 118Q cells slowed CAG repeat expansion in a dose-dependent manner that reflected its inhibition of MSH3/DNA interaction (Fig. 5).

While U2OS cells transduced with an exon 1 construct have provided a useful tool to dissect the molecular mechanisms underlying CAG repeat expansion, they may not reflect the full *in vivo* situation (i.e. somatic expansion in post-mitotic neurones in the HD brain). Therefore, we have looked at the role of the MSH3 IDL binding pocket in our HD iPSC 125Q-derived MSN-enriched cultures, a physiologically relevant cell model. These predominantly neuronal post-mitotic cultures (>95% βIII-tub+, Supplementary Fig. S4C and D) show increases in CAG repeat size over time in culture and have been used previously to study the role of MMR proteins in SI [28, 40]. We treated differentiated cultures with the small molecule CP1 and assessed target engagement and measured repeat size over 98 days (Fig. 5E–H). In agreement with the effect observed in U2OS cells, CP1 treatment reduced MSH3 CAG2 oligo binding and slowed expansion relative to control cells. No effects on cell morphology or viability were observed, indicating CP1 is well tolerated by the cells at this concentration (Supplementary Fig. S4B–D). However, long-term CP1 treatment did reduce MSH3 levels moderately. It is possible preventing normal DNA interactions with CP1 has a destabilizing effect on MutSβ in MSN-enriched cultures, leading to reduced MSH3 levels. The lowering of MSH3 levels complicates the interpretation of any effects CP1 has on expansion. Whilst MSH3 lowering is known to slow CAG repeat expansion, published data suggest that, to reduce expansion to the rates observed here, we would have to lower MSH3 levels by 83% [40]. Therefore, reduced MSH3 IDL binding is likely to be the predominant reason for the slowed repeat instability at 5 µM (Fig. 5G and H).

Showing this effect in differentiated neuronal cells is important because it demonstrates that CP1 is effective in slowing expansion of the *HTT* CAG repeat in non-dividing cells that express endogenous MSN DDR proteins. However, it should be noted that CP1 is not entirely specific and shows some binding to other proteins (notably PTGES2) [36]. Whilst none of the proteins have previously been implicated in CAG repeat expansion, we cannot rule out the observed slowing is mediated by one of these off-target interactions.

The data reported here show MSH3 interactions with IDLs through the Y254/K255 binding pocket play an important part in the expansion process in a U2OS HTT exon 1 118Q model. We also report on a small molecule targeting this site that slows repeat expansion in U2OS HTT exon 1 118Q cells and in HD patient stem cell-derived neurones. CP1 is a covalent modifier that shows toxicity at higher concentrations and some promiscuity in terms of target engagement [36] and therefore may not represent a great therapeutic candidate in itself. However, our data suggest that the IDL binding pocket of MSH3 may represent a viable therapeutic target worth future study, particularly as *MSH3* is relatively tolerant of loss-of-function variation in humans [1].

## Supplementary Material

ugag031_Supplemental_File

## Data Availability

The data are available in the article, in its online Supplementary data, or available upon request. To calculate modal CAG repeat length, GeneMapper data were exported and analysed with a custom R script, available at https://michaelflower.org/. HiFi reads were demultiplexed by dual-barcode assignment using Ophelia (https://github.com/mikeflower/ophelia, https://doi.org/10.5281/zenodo.18888576) and analysed using the Duke pipeline (https://github.com/mike-flower/duke, https://doi.org/10.5281/zenodo.18888560).
