# Supplementary material for: Genetic or pharmacological disruption of the MSH3 Y245/K246 IDL binding pocket slows CAG repeat expansion
Source: NAR Mol Med. 2026 Jun 5;3(2):ugag031. doi: 10.1093/narmme/ugag031 (PMC13273312; doi:10.1093/narmme/ugag031)
Supplement: ugag031_Supplemental_File [file ugag031_supplemental_file.docx]

Supplementary data

Supplementary figures


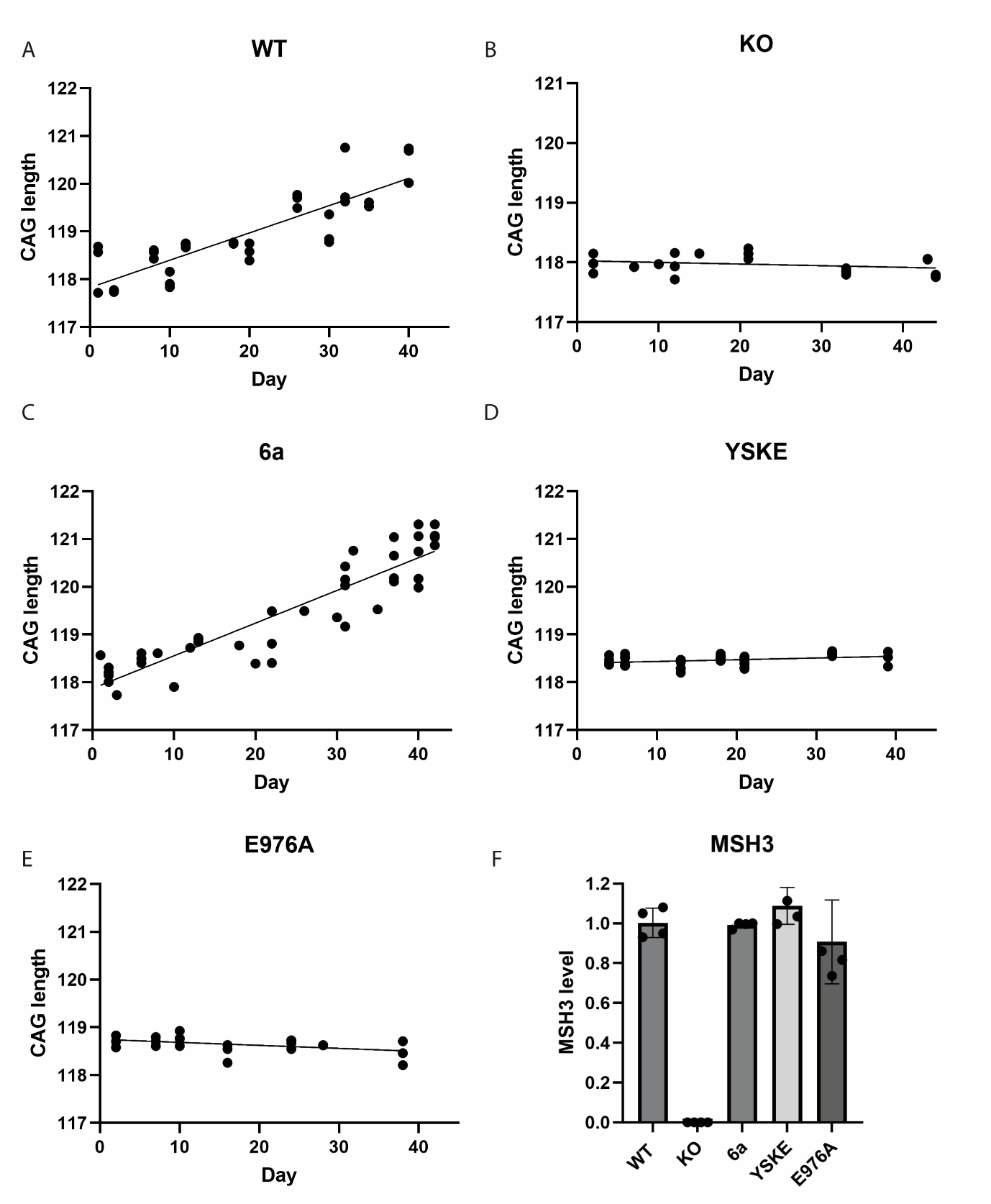


**Figure S1. Genetic blockade of the MSH3 IDL binding pocket disrupts MMR function**

**(A-E)** U2OS WT or MSH3 KO cells or KO cells complemented with myc MSH3 6a, YSKE or E976A constructs were transduced with LV *HTT* exon 1 118 CAG. Repeat size was monitored over 40 days in culture using fragment analysis. Scatter plots showing each data point included in Figure 1 are shown, along with a simple linear regression line fitted to the data. **(F)** Histogram showing expression of MutS proteins in WT, MSH3 KO and KO cells complemented with myc MSH3 6a, YSKE or E976A constructs. Complemented cells were treated with 0.1 ng/ml dox except the E976A line in which 1 ng/ml dox was used. Quantification of Western blots from two lines of each genotype measured in duplicate are shown (mean ± sd). No significant differences in MSH3 expression levels between WT and complemented cells were seen.

**
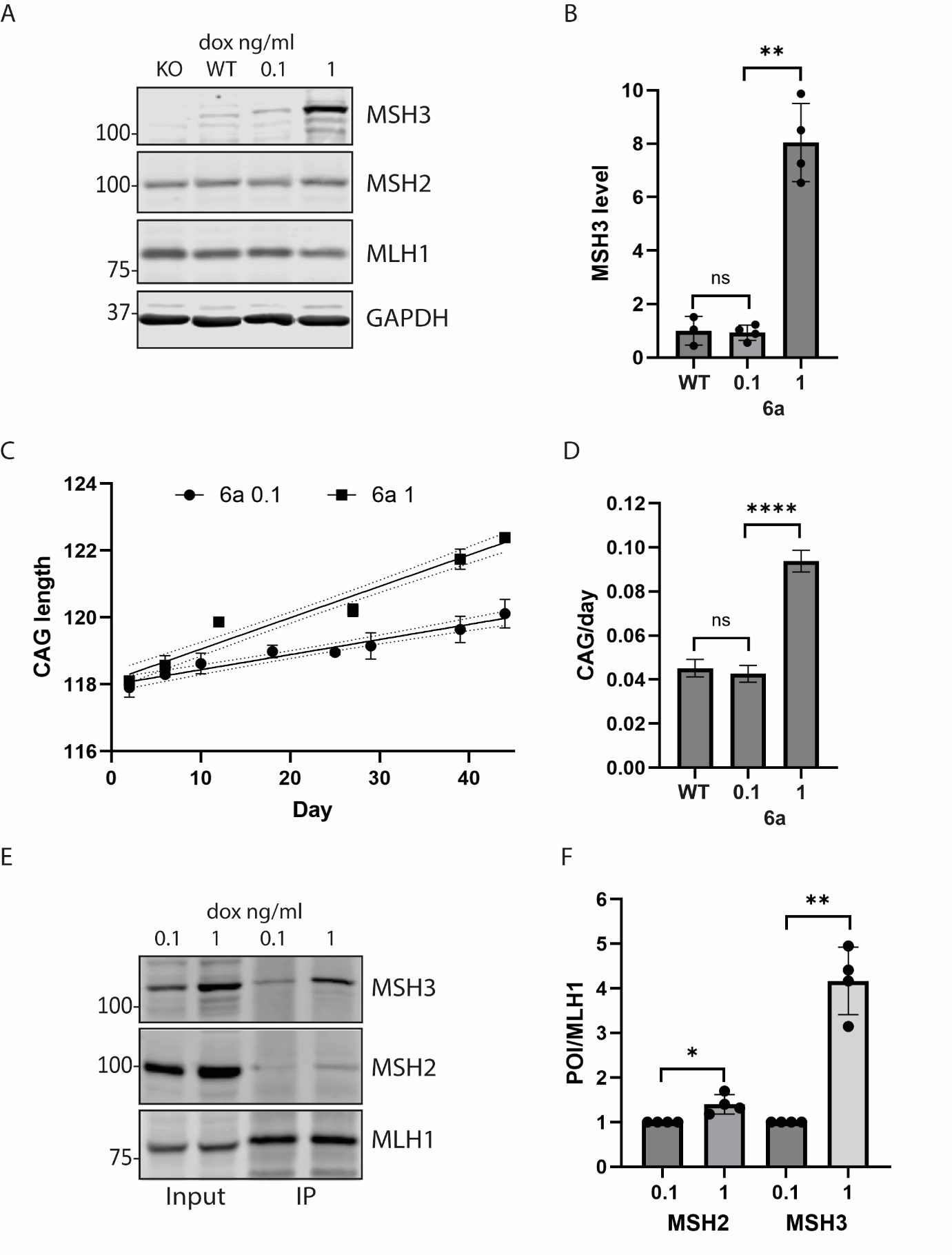
**

**Figure S2 MSH3 overexpression speeds up CAG repeat expansion**

(**A**) Immunoblots from lysates of U2OS MSH3 KO, WT or KO cells complemented with myc MSH3 6a and treated with the indicated concentrations of dox. myc MSH3 6a expression close to endogenous levels is induced by 0.1 ng/ml dox, increasing dox concentration to 1 ng/ml leads to MSH3 overexpression (~8 fold). (**B**) Quantification of blots normalized to U2OS WT is shown in the histogram. Data from two independently derived MSH3 KO cell lines complemented with myc MSH3 6a, each measured twice is shown (mean ± sd, ns = non-significant, ** p<0.01). (**C**) MSH3 KO cells complemented with myc MSH3 6a construct were transduced with LV *HTT* exon 1 118 CAG. Following transduction 0.1 or 1 ng/ml dox was added to the media and repeat size was monitored over 40 days in culture using fragment analysis. CAG repeat expansion is rescued by the MSH3 6a construct expressed at similar levels to the endogenous protein (6a 0.1). Cells over-expressing myc MSH3 6a drive CAG repeat expansion above the rate seen in WT cells (6a 1). Expansion assays from two independently derived MSH3 KO cell lines complemented with myc MSH3 6a were each run in parallel. Data from these four expansion assays for each condition are combined for each curve. (**D**) Histogram shows regression analysis of expansion curves in panel C. Note myc MSH3 overexpression leads to expansion rates above that seen in WT cells (mean ± standard error, ns = non-significant, **** p<0.0001). (**E**) Immunoblots showing anti-MLH1 IP’s from U2OS extracts made from myc MSH3 6a cells treated with 0.1 or 1 ng/ml dox concentrations, yielding the equivalent of endogenous expression or 8-fold myc MSH3 6a overexpression. Note myc MSH3 6a overexpression leads to an increase in MSH2 levels and a larger increase in MSH3/MSH2 (MutSβ) co-immunoprecipitated with MLH1. **(F)** Quantification of MSH2/MLH1 or MSH3/MLH1 in the IP fractions normalized to 0.1 ng/ml dox is shown in the histogram (Data from duplicate IPs from two independently derived MSH3 KO cell lines complemented with myc MSH3 6a, mean ± sd, * p<0.05, ** p<.001).

**
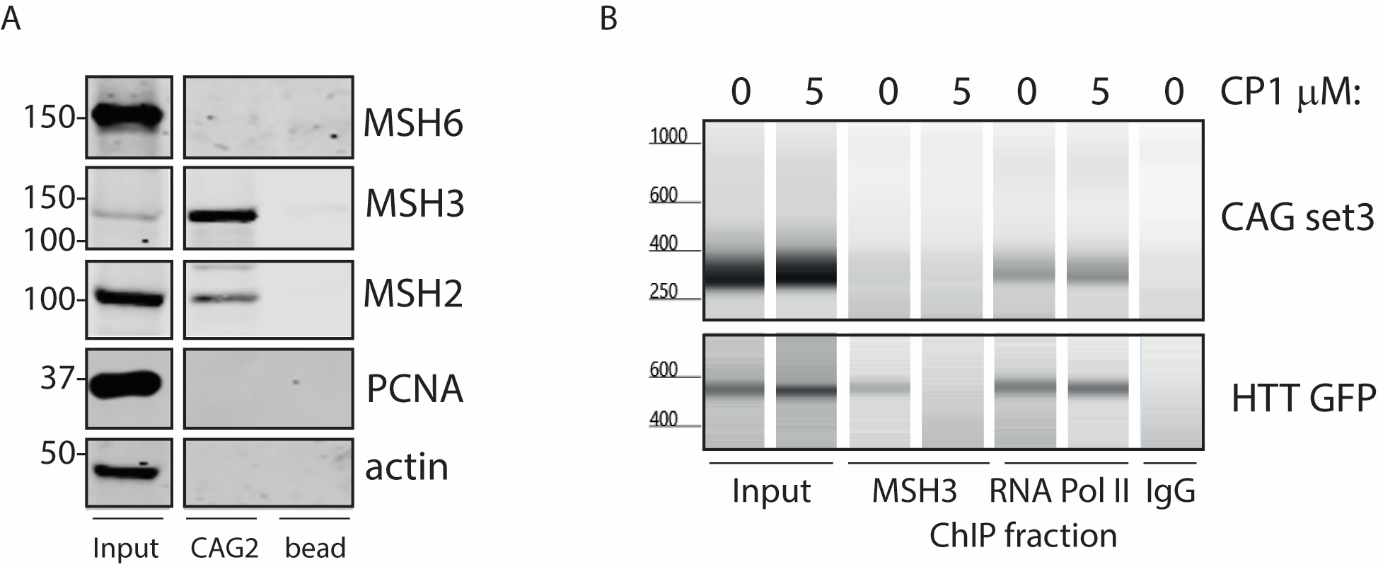
**

**Figure S3 MSH3 DNA interactions**

(**A**) U2OS WT cell extracts were prepared and incubated O/N with CAG2 oligos immobilized on streptavidin magnetic beads. Beads were isolated and washed with a magnetic device. Input (5%) and eluted proteins (CAG2) were immunoblotted with the indicated antibodies. Unconjugated beads (bead) were used as controls. (**B**) FAN1 KO cells transduced with LV HTT exon 118Q were treated with CP1 5 µM (5) or vehicle (0). Cell extracts were prepared for ChIP analysis and immunoprecipitated with anti MSH3, RNA PolII or control non-specific mouse IgG antibodies. DNA from input and ChIP fractions was purified and probed with primers targeting the *HTT* exon 118Q CAG repeat present in LV HTT exon 118Q (CAG set 3) or GFP primers downstream from this in the insertion cassette (HTT GFP). Input (5%) and ChIP fractions were analysed using TapeStation apparatus and software. Although DNA levels in MSH3 ChIP fractions were low, CP1 treatment consistently reduced DNA levels in the MSH3 ChIP fractions


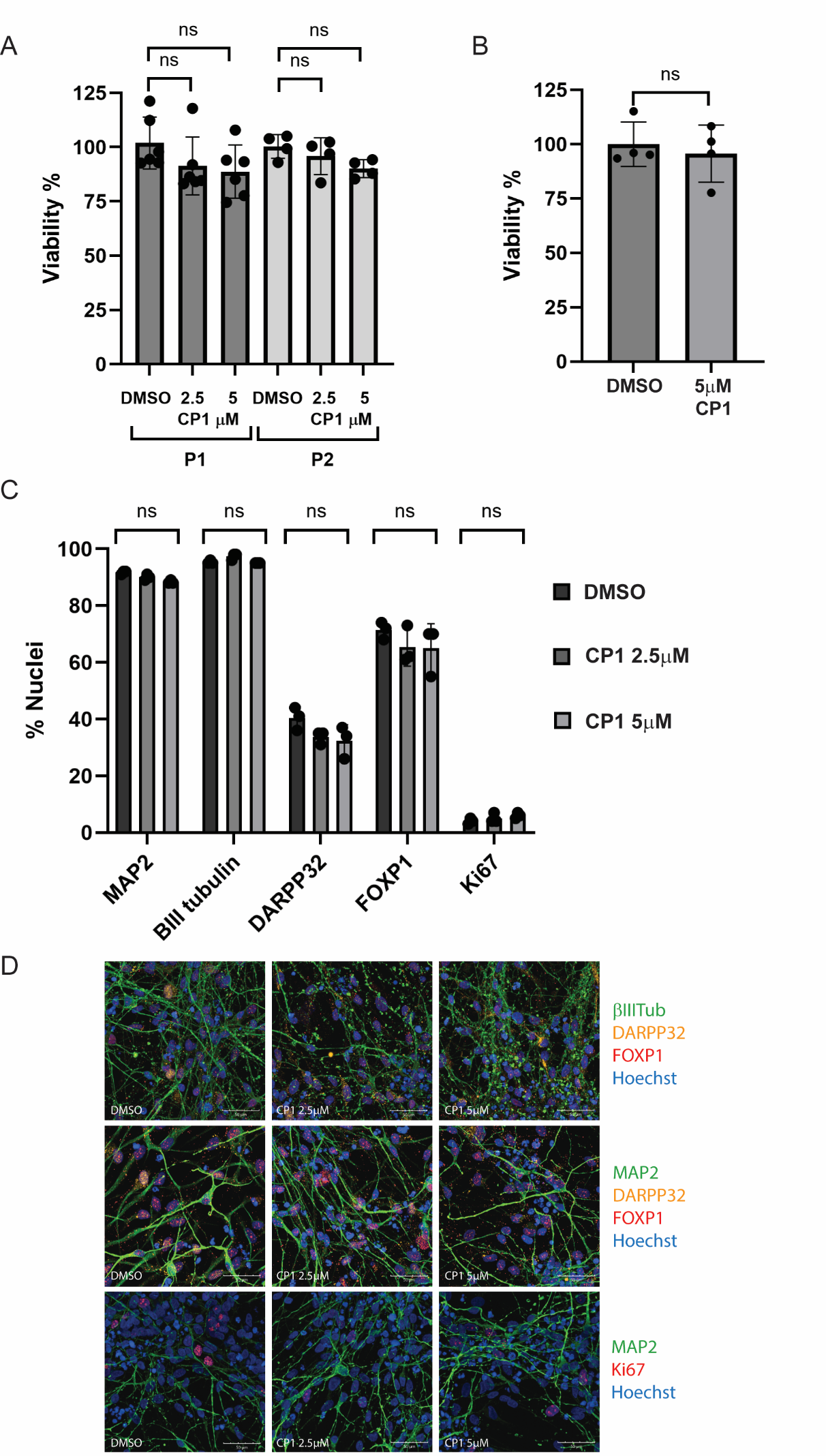


**Figure S4 CP1 toxicity to U2OS and iPSC 125Q derived MSN enriched cultures**

(**A**) MTT assay showing viability of U2OS WT cells across two cell passages (P1 and P2) following vehicle (DMSO) 2.5 µM or 5 µM CP1 treatment (mean ± sd, ns = non-significant). (**B**) MTT assay showing viability in iPSC 125Q derived MSN enriched cultures following 84 days of vehicle (DMSO) or 5 µM CP1 treatment (mean ± sd, ns = non-significant). (**C**) Quantification of marker positive cell frequency for MAP2, βIII Tubulin, DARPP32, FOXP1 and Ki67 as proportion of the total cells counterstained with Hoechst following vehicle (DMSO), 2.5 µM or 5 µM CP1 treatment. CP1 treatment did not change culture content significantly (mean ± sd, ns = non-significant). (**D**) Representative immunocytochemistry of HD iPSC-derived MSN enriched cultures after 84 days of vehicle (DMSO), 2.5 µM or 5 µM CP1 treatment. Immunostaining with the indicated antibodies and counterstaining with Hoechst is shown.


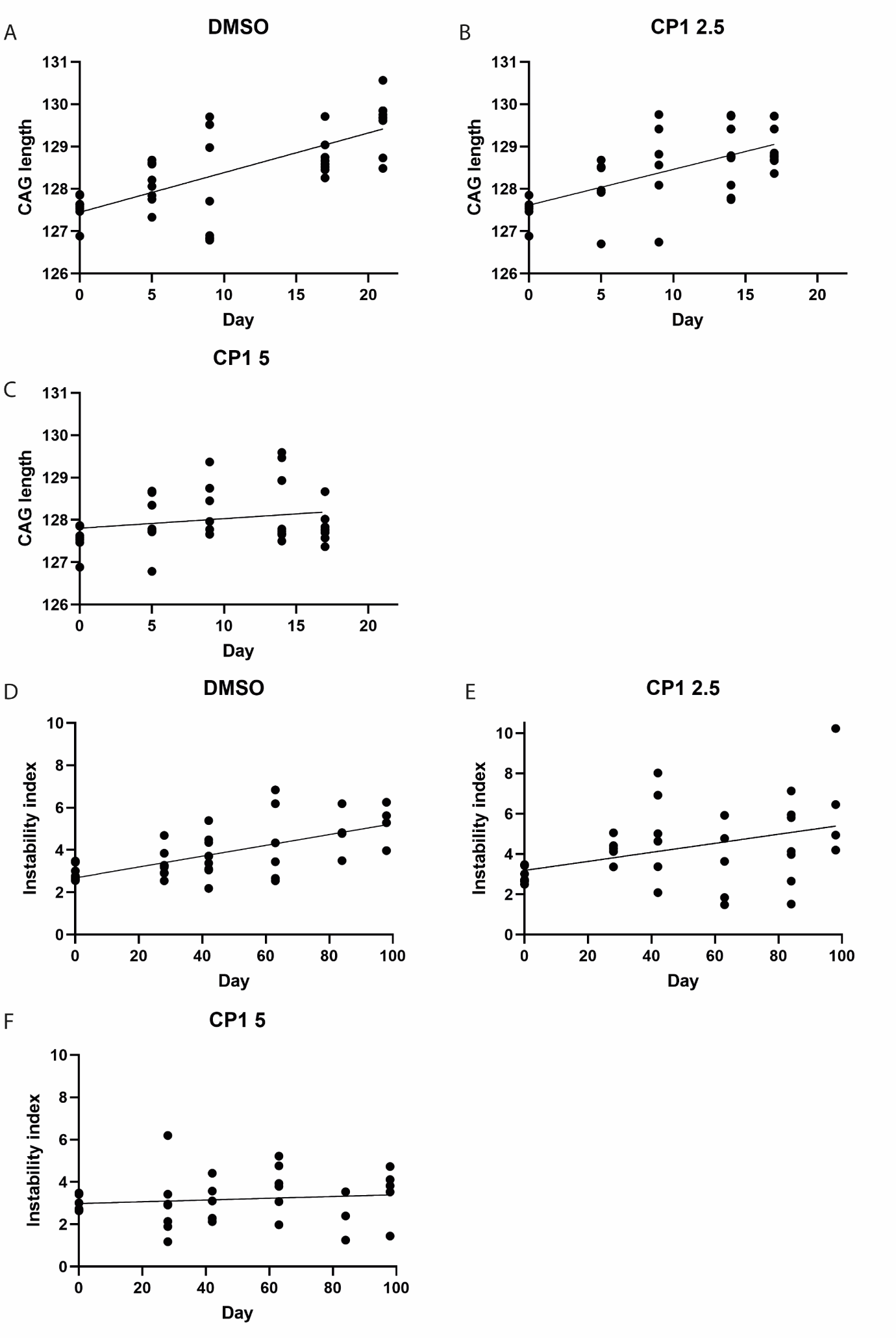


**Figure S5 Pharmacological inhibition of the MSH3 IDL binding pocket slows CAG repeat expansion**

U2OS FAN1 KO cells previously transduced with LV HTT exon 1 118 CAG were cultured in the presence of vehicle (DMSO) or CP1 at 2.5 µM or 5 µM (CP1 2.5 and 5 respectively). Repeat size was monitored over 17-21 days in culture using fragment analysis. Three independent expansion time courses were run in parallel for each condition and each was measured twice using fragment analysis. (**A-C**) Scatter plots showing every point used in the statistical analysis shown in Figure 5D and a simple linear regression line fitted to the data are shown. (**D-F**) Expansion assays showing HTT CAG instability indices in iPSC-derived MSN-enriched cultures treated from day 36 with vehicle (DMSO) or CP1 at 2.5 µM or 5 µM (CP1 2.5 and 5 respectively). Three independent expansion time courses were run in parallel for each condition and each was measured twice using long-read repeat sizing of the endogenous HTT CAG repeat. Scatter plots showing every point used in the statistical analysis shown in Figure 5H and a simple linear regression line fitted to the data are shown.


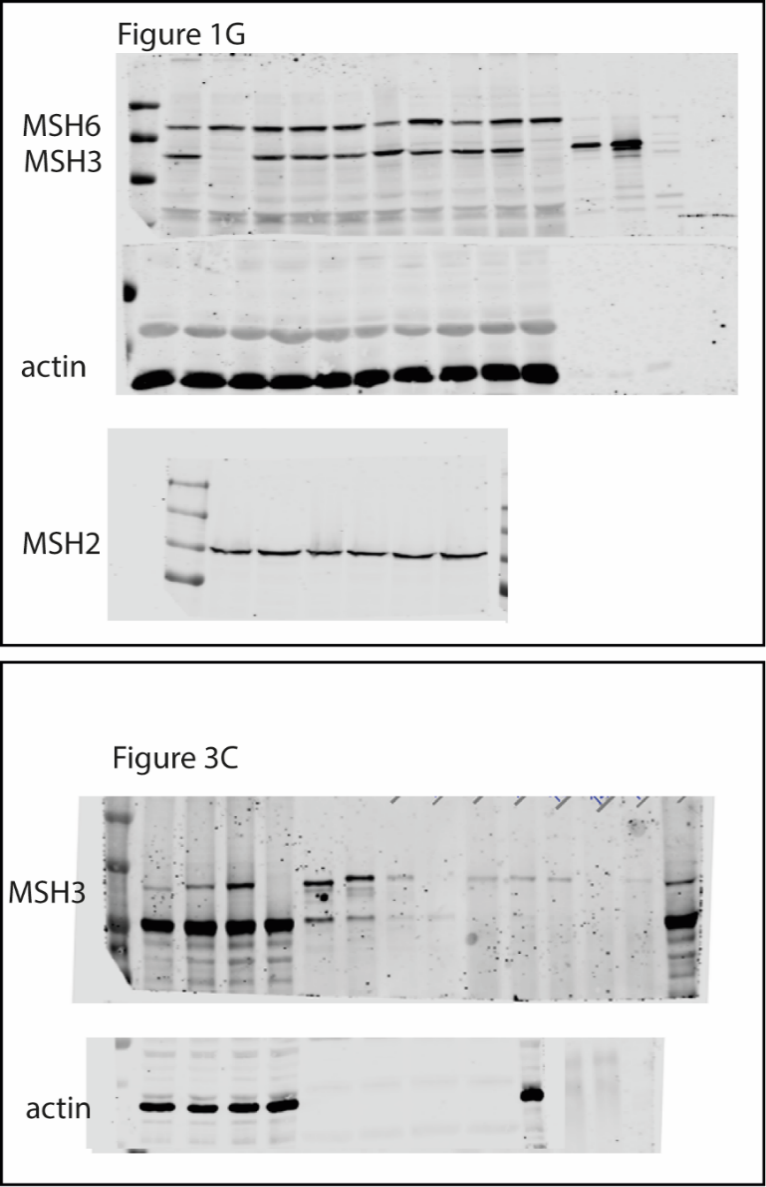


**Figure S6 Uncropped blots for Figures 1G and 3C in main text**

Figure numbers/panels are indicated along with the antibodies used to probe the blots
